# The Future of Pig Liver Xenotransplantation

**DOI:** 10.3389/ti.2025.13622

**Published:** 2025-07-18

**Authors:** Ahmad Karadagi, Gabriel C. Oniscu

**Affiliations:** ^1^ Division of transplantation surgery, Department of Clinical Science, Intervention and Technology, Karolinska Institutet, Stockholm, Sweden; ^2^ Department of Transplantation, Karolinska University Hospital, Huddinge, Sweden

**Keywords:** liver transplant, xenotransplantation, cross circulation, auxiliary liver transplantation, decedent model

## Abstract

Recent technological advances in genetic engineering have led to a renewed interest in xenotransplantation. Milestones achievements in heart, kidney and more recently liver xenotransplantation raise the hope of an alternative source of organs and pave the way for first in human clinical trials. With these rapid developments, it is important to consider what may be the place of liver xenotransplantation in the treatment armamentarium and what are the challenges to achieve clinical liver xenotransplantation.

## Introduction

Liver transplantation is the curative treatment for end-stage liver failure of many etiologies. The limited availability of human organs suitable for transplantation is, however, a significant limiting factor worldwide. Therefore, heart, kidney and liver xenotransplantation, as an alternative source of organs has been explored with a focus on pigs as the primary species for such transplants due to their anatomical and physiological similarities to humans, and relatively permissive ethical considerations. Recent technological advances in genetic engineering have enabled reliable gene modifications to wild-type pigs to abrogate the known glycan xenoantigens (alpha-Gal, Neu5Gc, and SDa) responsible for preformed antibody mediated hyperacute rejection in humans and other primates [1]. Further introduction of multiple human transgenes has enabled the transplantation of hearts as reported by Griffith et al. into a living human [2]. Subsequently, kidneys and liver xenotransplantation into selected living human patients, under single patient expanded access authorization or compassionate use, have been undertaken and detailed peer-reviewed reports from these recent endeavours are greatly anticipated.

Regardless of the significant advancements in genetic engineering and improved surgical techniques, xenotransplantation of the liver shows inferior outcomes in nonhuman primate studies compared to other organs. Whilst the heart and kidney xenotransplantation survival is measured in years [3, 4], the longest survival of a pig liver-to-baboon is still 29 days as reported in 2017, with a requirement for continuous infusion of human coagulation factors [5]. The liver’s complex physiology and the multifaceted challenges of replicating or replacing its functions may have contributed to this discrepancy.

## Potential Roles for Liver Xenotransplantation

With these rapid developments, it is important to consider what may be the place of liver xenotransplantation in the treatment armamentarium and what are the challenges to achieve this. Whilst life-sustaining orthotopic liver xenotransplantation for chronic liver disease indications poses significant challenges that are yet to be overcome, a liver xenograft holds the potential to serve as a bridge to an allograft liver transplant. This could be undertaken either as a full organ transplant following native hepatectomy for well-defined scenarios or, more feasibly, as an auxiliary support in cases of acute failure of the native liver. In the latter case, auxiliary xenograft implantation of relatively small porcine livers will not interfere with the vascular supply of the native liver, would allow time for the native liver to regenerate adequately and resume its normal functions and would enable a swift subsequent removal of the bridging xenograft. However, the impact of a xenograft on the liver’s natural regenerative processes is yet unclear. It is uncertain whether the xenograft would inhibit the regeneration of the patient’s native liver or if it would itself experience growth. Indeed, both scenarios present complex challenges that require additional investigation.

The development of *ex vivo* perfusion devices offers a promising solution for the use of xenografts as liver support, removing the need for surgical implantation and acting as an external cross-circulation system that provides critical hepatic metabolic functions and synthesis of vital proteins including coagulation factors during the acute injury phase. This approach is particularly beneficial in cases of acute liver failure, where brief, temporary support is needed. It is yet unclear if the extracorporeal machine perfusion approach may also minimise cellular interaction between the xenograft and patient, thus perhaps reducing the risk of rejection.

## Current Status

Recently, several milestone achievements in the liver xenotransplantation field were reported. In January 2024, a team from the University of Pennsylvania (UPenn) announced the first use of extracorporeal xenograft liver circulation in a decedent human recipient following brain death. The recipient was connected to a genetically engineered pig liver, developed by eGenesis Inc., which overexpressed seven human transgenes in addition to knock-out of three glycan xenoantigens, and inactivated PERV elements through three rounds of editing and cloning. This was the same genetic background that was recently reported to maintain kidney xenografts for up to 2 years in a nonhuman primate model was used [4]. The OrganOx^®^ machine perfusion device sustained the xenograft, and over a 72-hour period, there was no evidence of rejection, as reported by the investigators to the news outlets [5] but the scientific report and evidence and the final assessment are yet to be published. The trial was electively concluded as planned. It is important to note that the recipient had an intact and functioning liver, which may blur the potential compatibility and function of porcine proteins in humans. Although the preliminary findings are promising and the proof of concept was demonstrated, particularly considering the significant challenges observed in nonhuman primate studies, the short duration of the trial means that further research is necessary to reach definitive conclusions with regards to functional support.

In March 2024, a team from Xijing, China announced the implantation of a genetically modified porcine liver containing six genetic modifications, that deleted three glycan xenoantigens and introduced three genes for human proteins, into a decedent recipient. The liver xenograft was observed for 10 days, and the group report no gross evidence of rejection. Two hours after portal vein reperfusion of the xenograft, bile production was noted. Porcine liver-derived albumin increased after surgery and alanine aminotransferase levels remained in the normal range. Blood flow velocity in the porcine hepatic artery and portal and hepatic veins remained at an acceptable level [6]. Similar to the UPenn report, the recipient liver was not removed, hindering a full evaluation of the pig liver functions. A combined liver-kidney transplant has also been undertaken in a decedent model in China, demonstrating the feasibility of the procedure although details are still awaited.

On 17^th^ of May 2024, a team from the First Affiliated Hospital of Anhui Medical University reported a liver xenotransplantation into a living human patient. The recipient, a 71-year-old man diagnosed with a liver tumour underwent right hemi-hepatectomy followed by an auxiliary liver xenotransplantation due to insufficient size of the remnant left liver. The pig liver had 10 genetic edits (three to delete the glycan xenoantigens and 7 to induce production of human proteins) engineered at the Yunnan Agricultural University in Kunming, China. Early reports of the patient’s recovery were favourable and further updates are awaited.

## Present Challenges and Prospects for Clinical Application

There remain numerous immunological barriers for all types of pig-to-human xenotransplantation and these are particularly important for liver transplantation. Although immediate hyperacute rejection has diminished with the advance of alpha-Gal knock-out pigs, many other factors contributing to graft failure remain.

The introduction of novel immunosuppressive drugs including anti-CD154 monoclonal antibodies has offered some benefits for the successful outcome of xenotransplantation as evidenced by pre-clinical animal studies [6, 7] and as such the clinical use of an anti-CD154 monoclonal antibody is likely to play a significant role in any future application of xenotransplantation.

Thrombocytopenia occurring without signs of a rejection reaction, indicates alternative mechanisms and further molecular incompatibilities. Compared to human livers, porcine livers have reduced albumin production [8]. Additionally, pig thrombomodulin does not interact correctly with human Protein C receptor. These are among multiple recognized incompatibilities being addressed by genetic engineering and introduction of human transgenes.

Unlike other organs such as the heart and kidneys, the liver has multiple complex functions, both detoxifying harmful compounds and synthesizing a wide range of proteins that play a vital role in both hepatic and systemic processes. While there are parallels between pig and human physiology, considerable differences exist. Conserved functions for detoxifying compounds absorbed via the enteric system may be similar, yet the exact metabolites produced by these processes may vary. Moreover, as seen in human liver pathology, abnormal folding or genetic mutations of proteins may lead to storage diseases and accumulation of faulty proteins causing injury both in the liver and elsewhere. These potential pathologies need to be considered and monitored, especially since they tend to manifest after several years of accumulation. These long-term effects are not apparent in animal studies and could not be assessed in the groundbreaking work undertaken so far. Thus, patient selection and a clearly defined anticipated benefit for the first in human trials are critical.

The genetic modifications in engineered pig organs are specifically optimized for compatibility with human immune systems, which suggests that data from nonhuman primates may not reflect the full potential anticipated in humans. Furthermore, this implies that the outcomes in humans may surpass the results seen in animal models.

Zoonotic transmission remains a major hurdle in xenotransplantation compounding the infectious complications already inherent to organ transplantation. Porcine cytomegalovirus has emerged as a difficult to test and treat virus in this context. While it may not infect the human recipient, it may injure the xenograft causing potential failure. This mechanism is believed to have contributed to the dysfunction of the first heart xenotransplantation.

Although still debated, xenogeneic organs, when transplanted into recipients, are expected not to provoke the production of alloreactive antibodies. Consequently, they appear to not sensitize the recipient for future allogenic transplantations. This is particularly important in auxiliary xenotransplantation, where liver xenotransplantation serves as a bridge to subsequent allograft transplantation [9, 10].

Xenotransplantation, including liver xenotransplantation represents a promising Frontier in transplantation medicine, offering hope for addressing the dire shortage of human donor organs. The field has witnessed significant progress, with the recent decedent studies and the successful report of a living human recipient marking a pivotal moment in its development.

Clinical trials may well be the next Frontier, but the challenges ahead are significant, including immunological barriers [11], longitudinal effects, regulatory restrictions and a clear definition of the foreseen role in the management of chronic or acute liver failure. To reassure the public and ensure safety, it is paramount that current genetic engineering and breeding are undertaken under DPF (Designated Pathogen-Free) conditions to ensure that donor pigs have a very high safety profile. Moreover, it is essential to maintain a dialogue with the public, ensuring that the societal implications of xenotransplantation are considered alongside the scientific advancements [12]. To achieve this, it is paramount that Europe re-engages in xenotransplantation and some of the clinical experimental work and indeed clinical trials are undertaken here.
